# Health inequalities of 57,541 prisoners in Korea: a comparison with the general population

**DOI:** 10.4178/epih.e2021033

**Published:** 2021-05-06

**Authors:** Seohyun Yoon, Young-Su Ju, Jaehong Yoon, Ji-Hwan Kim, Bokyoung Choi, Seung-Sup Kim

**Affiliations:** 1 Department of Public Health Sciences, Korea University, Seoul, Korea; 2 Ewha Medical Research Institute, Ewha Womans University School of Medicine, Seoul, Korea; 3 National Medical Center, Seoul, Korea; 4 Interdisciplinary Program in Precision Public Health, Korea University, Seoul, Korea; 5 Department of Social and Behavioral Sciences, Harvard T. H. Chan School of Public Health, Boston, MA, USA

**Keywords:** Correctional health, Prison health, Prison healthcare, Prison inmates, Korea

## Abstract

**OBJECTIVES:**

This study aimed to examine health disparities between prisoners and the general population in Korea.

**METHODS:**

We sought to estimate the prevalence of 17 physical and mental diseases using the nationwide medication prescription dataset among the total population of prisoners (n=57,541) in Korea. Age- and sex- standardized prevalence ratios (SPRs) were estimated to compare the disease prevalence between the prisoners and the general population. The disease prevalence for the general population was calculated from the prescription dataset for a representative of the Korean population (n=926,246) from the 2013 Korean National Health Insurance Service-National Sample Cohort. Furthermore, the prevalence of these diseases was compared between prisoners and a low-income segment of the general population (n=159,781).

**RESULTS:**

Compared to the general population, prisoners had higher prevalence of almost all physical and mental diseases, including hyperlipidemia (SPR, 20.18; 95% confidence interval [CI], 19.43 to 20.94), pulmonary tuberculosis (SPR, 9.58; 95% CI, 7.91 to 11.50), diabetes (SPR, 6.13; 95% CI, 5.96 to 6.31), cancer (SPR, 2.36; 95% CI, 2.07 to 2.68), and depression (SPR, 46.73; 95% CI, 44.14 to 49.43). When compared with the low-income population segment, higher prevalence were still found among prisoners for most diseases, including pulmonary tuberculosis (SPR, 6.39; 95% CI, 5.27 to 7.67) and depression (SPR, 34.71; 95% CI, 32.79 to 36.72).

**CONCLUSIONS:**

We found that prisoners were more likely to be unhealthy than the general population, even in comparison with a low-income segment of the general population in Korea.

## INTRODUCTION

Globally, there are more than 10 million people in prison, and this number is steadily increasing [1]. As of 2015, the United States (US) prison population was the world’s largest, with 2.2 million, followed by China with 1.6 million and Russia with 0.6 million [2]. In Korea, there are more than 54,000 prisoners, representing a rate of 106 per 100,000 based on an estimated national population of 51.17 million in 2019 [1,3].

Prisoners are one of the most vulnerable populations. Not only do they become marginalized through detention in prison and the attendant poor living conditions, but most also come from socioeconomically disadvantaged backgrounds [4-6]. Although prisoners have a constitutional right to health under international human rights law [7], previous studies have reported that prisoners’ access to healthcare and the quality of that care are often deficient [8,9]. Thus, they are more likely to have poor health status while in prison, which has the effect of widening the health inequality gap in society [5,6,10]. Furthermore, the health of prisoners can ultimately have a considerable influence on society, including the increased spread of infectious diseases or substantial financial burdens when they return to the community. Thus, it is necessary to pay attention systematically to the health of prisoners during their prison stay.

Many studies have consistently shown that prisoners have poorer health status than the general population [4,11-14]. Two studies in the US and Mexico reported that prisoners had a higher prevalence of several infectious and chronic diseases, including hepatitis, hypertension, diabetes, angina, myocardial infarction, and cancer than representative general population samples [15,16]. A review of 62 studies gathered from 12 developed countries found that prisoners were more likely to have a mental disorder than the general population, suggesting that typically about 1 in 7 prisoners have psychotic illnesses and/or major depression [17].

However, the previous literature regarding health disparities of prisoners has the following 2 limitations. First, few studies conducted to quantify the health disparities between prisoners and the general population using nationwide data. For example, a study in Japan showed that the incidence of tuberculosis among prisoners compared with the general population using annual reports of correctional statistics [18]. Second, most previous studies assessed disease prevalence based on datasets collected by self-reporting [19-21]. Considering that prisoners are not readily able to visit doctors, it seems probable that certain conditions are underreported in surveys.

To fill these gaps in knowledge, this study aimed to explore health disparities between prisoners and the general population in Korea, comparing the prevalence of 17 physical and mental diseases using a medication prescription dataset between the prisoners and the general population of Korea. Furthermore, we additionally compared the prevalence of these conditions between prisoners and a low-income segment of the general population because perisoners are more likely to have socioeconomically disadvantaged backgrounds [4-6].

## MATERIALS AND METHODS

### Data sources and study population

In Korea, about 58,000 people were imprisoned in 53 correctional facilities in 2016. The Korean government operates 52 correctional facilities under 4 regional corrections headquarters in Seoul, Daegu, Daejeon, and Gwangju. About 300 prisoners are housed at 1 additional facility, which has been privately run since the end of 2010. This study included a total population of 57,541 prisoners in all 52 government correctional facilities. Data were drawn from official prison records, which were collected on September 30, 2016 by the Korea Ministry of Justice and publicly reported for the first time in a report of the National Human Rights Commission of Korea [22].

We compared prison data with a large nationally representative administrative dataset from the National Health Insurance Service-National Sample Cohort (NHIS-NSC), a population-based cohort established by the Korean National Health Insurance Service (NHIS). The NHIS for all citizens was initiated in 1963 according to the National Health Insurance Act in Korea. Currently, the NHIS maintains and manages all health service utilization in Korea. The detailed structure and function of NHIS have been described elsewhere [23]. The NHIS-NSC comprised 2.2% of the total eligible Korean population in 2002, and participants were followed until 2013. We used a dataset obtained in 2013 from the NHIS-NSC in Korea. This dataset contained 1,014,730 individuals, of whom we included 926,246 individuals in our study after excluding participants who were < 10 years old because this population does not exist in prisons.

Additionally, considering that prisoners are more likely to have socioeconomically disadvantaged backgrounds, we compared the prevalence of diseases between prisoners and the low-income general population. The study population of NHIS-NSC was divided into 3 groups: (1) National Medical Aid beneficiaries, (2) the self-employed insured group, and (3) the employed insured group. National Medical Aid beneficiaries were defined as eligible recipients under the Medical Care Assistance Act, which was enacted to contribute to the improvement of national health and the enhancement of social welfare by providing medical benefits to indigent people (e.g., recipients of medical benefits under the National Basic Living Security Act and disaster victims in Korea), similar to Medicaid in the US. The latter 2 groups were sub-classified into 10 segments according to their income level which was based on health insurance premium [23]. There was no overlap between the 3 groups (self-employed insured, employed insured, and National Medical Aid beneficiaries). For the additional analysis, the low-income general population was defined as the lowest 2 segments of income level (0-20%) and those who were National Medical Aid beneficiaries. Consequently, the low-income population group consisted of 159,781 individuals.

### Disease prevalence

Disease prevalence was measured based on prescribed medications for each disease, both among prisoners and the general population. The investigated diseases were physical diseases: (1) hyperlipidemia, (2) myocardial infarction, (3) pulmonary tuberculosis, (4) viral hepatitis, (5) diabetes, (6) lumbar sprain, (7) angina pectoris, (8) hypertension, (9) cancer, (10) lumbar disc herniation, (11) cerebral infarction, (12) cerebral hemorrhage, (13) fracture, and (14) pneumonia; and mental diseases: (15) depression, (16) schizophrenia, and (17) insomnia.

There were at least 1 doctor and nurse in each prison of Korea [22]. For the mental diseases that require specialized services not normally offered in prisons, designated psychiatrists visit the prison regularly. When sick, prisoners apply to visit the prison doctor’s office to get medical treatment. If prison doctors determine that they cannot provide adequate treatment, prisoners visit a hospital outside the prison to get proper treatment, accompanied by several prison officers. Both in prison and at outside hospitals, the prison administrative system registers when prisoners are prescribed a medicine, with the name of the diagnosed disease, and documents how many prisoners have taken a prescribed medication. As the information reported on the prison system was only published for 1 day, specifically September 30, 2016, disease prevalence was calculated by dividing the number of prisoners who had taken a prescribed medicine by the total population of prisoners on that date. This included the number of prisoners who were prescribed the corresponding medications previously and had been taking them until that day, as well as those who were newly prescribed the medications on that day.

Likewise, disease prevalence in the general population was also measured based on prescribed medication data from the NHIS. Koreans are covered by health insurance when they receive medical care and buy prescription medications, and data are recorded in the NHIS with a specific diagnosis code. The NHIS uses disease classification codes from the Korean Classification of Diseases, sixth edition (KCD-6). Based on prison data, we defined individuals with specific diseases in the general population as those who bought doctor-prescribed medications with specific KCD codes (Supplementary Material 1). Unlike the prison data, the NHIS data do not provide information on how many individuals in the general population have taken a prescribed medication; instead, it only provides information on how many individuals were newly prescribed medication on a given day. In order to make the data comparable, we used prescription information from 14 days (September 24, 2013 to October 7, 2013) to calculate the prevalence in the general population.

### Statistical analysis

Indirect standardization was performed to assess whether the disease prevalence in the prisoners differed from that in the general population when prisoners’ age and sex were taken into account. The observed number of cases among prison population was compared with the expected cases based on age- and sexspecific prevalence of the general population. The results are presented as standardized prevalence ratio (SPRs) with 95% confidence intervals (CIs) [24]. All statistical analyses were performed with Stata/SE version 13.0 (StataCorp, College Station, TX, USA).

### Ethics statement

The study was conducted using previously collected de-identified data that are freely available; hence, it was deemed exempt by the Korea University Institutional Review Board (KU-IRB-17-EX-267-A-1).

## RESULTS

Table 1 shows the sex- and age-specific distribution of the prison and the general population. As of September 30, 2016, a total population of 57,541 prisoners was incarcerated in 52 prisons in Korea. The vast majority of prisoners (n= 53,767, 93.4%) were males. Most of the prisoners (74.6%) were in the 30-59 years age group, while 1.6% were in the ≥ 70 years age group (Table 1).

The prevalence and age- and sex-adjusted SPRs for each disease are shown in Table 2. Among 57,541 total prisoners, 8,286 (14.40%) had hypertension, 4,868 (8.46%) had diabetes, and 2,764 (4.80%) had hyperlipidemia. The prevalence of mental diseases, including depression (2.11%) and insomnia (1.22%), was generally higher than those of general population.

Not surprisingly, the prevalence of almost all diseases was significantly higher among prisoners than expected based on the age- and sex-specific prevalencein the general population (SPR> 1.00). For example, prisoners had 18.45- 46.73 times higher prevalence for mental diseases, including depression (SPR, 46.73; 95% CI, 44.14 to 49.43), schizophrenia (SPR, 40.91; 95% CI, 37.22 to 44.87), and insomnia (SPR, 18.45; 95% CI, 17.11 to 19.87), than the general population. Higher prevalence for several other diseases were also observed among prisoners than among the general population, including hyperlipidemia (SPR, 20.18; 95% CI, 19.43 to 20.94), pulmonary tuberculosis (SPR, 9.58; 95% CI, 7.91 to 11.50), diabetes (SPR, 6.13; 95% CI, 5.96 to 6.31), and cancer (SPR, 2.36; 95% CI, 2.07 to 2.68). Even when compared to the low-income population, the trend for higher prevalence among prisoners was still present for most diseases, including hyperlipidemia (SPR, 20.18; 95% CI, 19.43 to 20.94), pulmonary tuberculosis (SPR, 6.39; 95% CI, 5.27 to 7.67), and depression (SPR, 34.71; 95% CI, 32.79 to 36.72) (Table 2).

## DISCUSSION

The present study showed that the overall health of prisoners in Korea was poorer than that of the general population. Compared to the general population, prisoners had higher prevalence for 17 diseases, including hyperlipidemia, pulmonary tuberculosis, cancer, and depression; these findings were observed regardless of whether diseases were physical, mental, acute, or chronic. These observations are consistent with previous international studies on the health disparities of prisoners [4,11-16,18,19,25-27].

The consistent results across the diseases are quite understandable considering living conditions in prisons. Prisoners share close living spaces with others, including cells, showers, hygiene facilities, and recreation areas. This may not only cause a great deal of stress to prisoners, but also provide fertile grounds to transmit disease directly. The more overcrowded the prison, the greater those negative effects on prisoners’ health will be [28]. Korea is among the many countries where prison overcrowding is severe. According to Organization for Economic Cooperation and Development (OECD) statistics in 2016, facility capacities in Korea are exceeded by 120%, corresponding to the second-highest rate among OECD countries following Hungary (131%) [29]. This information provides useful context for understanding our findings of disproportionately high prevalence of infectious diseases among prisoners; for example, the prevalence of pulmonary tuberculosis and viral hepatitis was about 9.58 times and 7.27 times higher among prisoners than in the general population, respectively. Likewise, it is plausible that the prevalence of every measured mental disease among prisoners was well over 10 times higher than in the general population.

However, another findings that merits consideration is that the higher prevalence of disease among prisoners may be related to the medical environment of prisons. Although every prison in Korea has 1 or more attending doctors inside the prison, there is a limited number of doctor-hours available per day to fill the demand; it is not easy for prisoners to get timely and appropriate medical care in prison [14]. Prisoners usually have to wait for long times in queue before they can see a doctor, and sometimes their requests for healthcare services are rejected by prison officers if the symptoms do not seem to be severe. Further, when prisoners need to see a hospital doctor outside the prison for getting necessary medical treatment, waiting times may become even longer. This might negatively influence on prisoners’ health by increasing stress, exacerbating the disease onset and ultimately increasing the severity of illness [30,31]. This can be a crucial issue, particularly for prisoners with mental diseases requiring specialized services that cannot be attended to by general practitioner doctors in prison. This should be considered in the context of our findings that the prevalence of both major depression and schizophrenia was more than 40 times higher among prisoners than among the general population.

There might be a possibility that prisoners could have poor health conditions than the general population due to their deprived backgrounds before imprisonment [4-6,32]. For example, according to the 2011–2012 National Inmate Survey by the Bureau of Justice Statistics in the US, among those who reported ever having a chronic condition, more than 70% of both prisoners and jail inmates reported that they had a chronic condition at admission [33]. One study in Korea also found that the lowest-income group showed a significantly higher 3-year cancer mortality rate than the highest-income group [34]. However, our study showed that prisoners still had a higher prevalence of most diseases when we compared disease prevalence with the low-income population. It is necessary to examine the risk factors which influence poorer health status of prisoners even compared with a low-income segment of the general population in future studies.

### Limitations and strengths

Several limitations of this study should be noted. First, the age distribution within the ≥ 70 years age group might differ between prisoners and the general populations. Because of the limited data available on prisoners, we could not fully determine the details of the full age range above 70 years old. If the gap of the distribution of ages within the ≥ 70 years age group between 2 populations is large, the expected number of prisoners with diseases in this age group may be biased. Second, we could not exclude the possibility that SPRs might have been overestimated or underestimated due to differences in measurement of diseases prevalence between prisoners and general population. For a given date, the data for the general population only includes those who had been newly prescribed the medication on that particular day. In the prison dataset, however, on a given day, it was not possible to differentiate between those who were newly prescribed a medication from those who were currently taking the medication but were prescribed it previously. To minimize the possibility of SPR overestimation, we calculated the prevalence in the general population from 14 days of data, specifically choosing 7 days before and after the date on which we calculated the prevalence of the prisoners. Furthermore, we used data from the same month and the date(s) in prison and the general population datasets to consider seasonal effects, but the 3-year difference in the timing of data collection is nonetheless a limitation. As a final limitation, there might be overprescription in prison. According to a report on the medical status of detention facilities in Korea, prison doctors responded that more than half of their patients are being over-treated [35]. The difference in prescription between prison and general population could occur differential misclassification of measuring diseases.

Despite these limitations, this study also has strengths. To date, no health information on Korean prisoners has been published using total population data. To our knowledge, this is the first study to describe the health status of prisoners in Korea. Further, we obtained information on the general population from a large, nationally representative dataset to compare disease prevalence with the total population of prisoners. Additionally, because we used administrative record data for both prisoners and the general population, this study is less vulernable to recall bias compared to previous study using survey data. Finally, we additionally compared disease prevalence between prisoners and a low-income segment of the general population to consider prisoners’ background before imprisonment.

Until now, reports on the health of prisoners in Korea have been rare, despite a large and growing prisoner population. The findings of this study highlight that Korean prisoners have a higher prevalence of several physical and mental diseases than not only the general population, but the low-income segment of the population. Future studies need to identify the possible and modifiable factors which influence the health status of prisoners, for interventions to improve health outcomes in prison population.

## Figures and Tables

**Table 1.** Age distribution of study population by sex

Age (yr)	Total		Male		Female	
	Prison population	General population^1^	Prison population	General population^1^	Prison population	General population^1^
10-19	839 (1.5)	124,284 (13.4)	803 (1.5)	65,314 (14.1)	36 (1.0)	58,970 (12.7)
20-29	8,052 (14.0)	131,922 (14.2)	7,664 (14.3)	68,823 (14.9)	388 (10.3)	63,099 (13.6)
30-39	12,398 (21.5)	155,953 (16.8)	11,673 (21.7)	79,526 (17.2)	725 (19.2)	76,427 (16.5)
40-49	16,072 (27.9)	176,668 (19.1)	15,032 (28.0)	89,769 (19.4)	1,040 (27.6)	86,899 (18.7)
50-59	14,526 (25.2)	159,483 (17.2)	13,478 (25.1)	80,399 (17.4)	1,048 (27.8)	79,084 (17.0)
60-69	4,707 (8.2)	89,336 (9.6)	4,262 (7.9)	43,260 (9.4)	445 (11.8)	46,076 (9.9)
≥70	947 (1.6)	88,600 (9.6)	855 (1.6)	34,561 (7.5)	92 (2.4)	54,039 (11.6)
Total	57,541 (100)	926,246 (100)	53,767 (93.4)	461,652 (49.8)	3,774 (6.6)	464,594 (50.2)

Values are presented as number (%).

1 Korean nationwide random sample cohort.

**Table 2.** SPRs of health outcomes between the prison population and the general population in Korea

Diseases		Prison population (n=57,541)	General population (n=926,246)	Age- and sex- SPR (95% CI)	Low-income population^1^ (n=159,781)	Age- and sex- SPR (95% CI)
		Prevalence, n (%)	Prevalence, n (%)		Prevalence, n (%)	
Physical						
	Hyperlipidemia	2,764 (4.80)	2,735 (0.30)	20.18 (19.43, 20.94)	530 (0.33)	20.18 (19.43, 20.94)
	Pulmonary tuberculosis	115 (0.20)	175 (0.02)	9.58 (7.91, 11.50)	35 (0.02)	6.39 (5.27, 7.67)
	Viral hepatitis	554 (0.96)	795 (0.09)	7.29 (6.70, 7.92)	137 (0.09)	6.60 (6.06, 7.17)
	Diabetes	4,868 (8.46)	12,631 (1.36)	6.13 (5.96, 6.31)	2,758 (1.73)	4.96 (4.82, 5.10)
	Myocardial infarction	263 (0.46)	731 (0.08)	5.60 (4.94, 6.31)	163 (0.10)	4.78 (4.22, 5.40)
	Lumbar sprain	622 (1.08)	1,827 (0.20)	5.41 (4.99, 5.85)	364 (0.23)	5.65 (5.22, 6.12)
	Hypertension	8,286 (14.40)	32,650 (3.52)	4.56 (4.47, 4.66)	6,930 (4.34)	4.40 (4.30, 4.49)
	Angina pectoris	401 (0.70)	1,672 (0.18)	4.72 (4.27, 5.20)	381 (0.24)	4.01 (3.63, 4.42)
	Cancer	238 (0.41)	2,254 (0.24)	2.36 (2.07, 2.68)	421 (0.26)	2.13 (1.86, 2.41)
	Lumbar disc herniation	348 (0.60)	2,855 (0.31)	2.26 (2.03, 2.51)	685 (0.43)	1.65 (1.48, 1.83)
	Cerebral hemorrhage	34 (0.06)	288 (0.03)	2.00 (1.39, 2.79)	85 (0.05)	0.85 (0.59, 1.19)
	Cerebral infarction	139 (0.24)	1,589 (0.17)	1.96 (1.65, 2.31)	373 (0.23)	1.48 (1.24, 1.75)
	Fracture	155 (0.27)	1,794 (0.19)	1.94 (1.64, 2.27)	412 (0.26)	1.50 (1.28, 1.76)
	Pneumonia	40 (0.07)	740 (0.08)	1.18 (0.84, 1.60)	175 (0.11)	0.91 (0.65, 1.24)
Mental						
	Depression	1,215 (2.11)	692 (0.07)	46.73 (44.14, 49.43)	144 (0.09)	34.71 (32.79, 36.72)
	Schizophrenia	450 (0.78)	158 (0.02)	40.91 (37.22, 44.87)	44 (0.03)	20.45 (18.61, 22.43)
	Insomnia	701 (1.22)	960 (0.10)	18.45 (17.11, 19.87)	217 (0.14)	12.09 (11.21, 13.02)

SPR, standardized prevalence ratio; CI, confidence interval.

1 Individuals who belong to the lowest 20% of household income level or who are Medical Aid beneficiaries.
