# Supplementary material for: Health inequalities of 57,541 prisoners in Korea: a comparison with the general population
Source: Epidemiol Health. 2021 May 6;43:e2021033. doi: 10.4178/epih.e2021033 (PMC8289480; doi:10.4178/epih.e2021033)
Supplement: Supplementary file 1 [file epih-43-e2021033-suppl.docx]

Supplementary Material 1. Diagnosis codes used to define diseases in the general population from Korean National Health Insurance Service-National Sample Cohort data using the Korean Classification of Disease, sixth edition^1^

|  | **Disease** | **Diagnosis code** |
| --- | --- | --- |
| ***Physical*** | | |
|  | Hyperlipidemia | E78.0–E78.5 |
|  | Myocardial infarction | I21–I22, I24–I25 |
|  | Pulmonary tuberculosis | A15–A16 |
|  | Viral hepatitis | B15–B19 |
|  | Diabetes | E10–E14 |
|  | Lumbar sprain | S33.5–S33.7 |
|  | Angina pectoris | I20 |
|  | Hypertension | I10–I15 |
|  | Cancer | C00–C97 |
|  | Lumbar disc herniation | M51.0–M51.1 |
|  | Cerebral infarction | I63 |
|  | Cerebral hemorrhage | I60–I62, S06.4–S06.6 |
|  | Fracture | M80, M84.4, M90.7, M49.5, M84.3, M48.4, M 96.6, S02, S12, S22, S32, S42, S52, S62, S72, S82, S92, T02, T08, T10, T12, T14.2 |
|  | Pneumonia | J12–J18 |
| ***Mental*** | | |
|  | Depression | F32–F33 |
|  | Schizophrenia | F20 |
|  | Insomnia | G47.0, F51.0 |
| ^1^Modified version of the International Classification of Diseases, 10th Revision for the Korean healthcare system. | | |
